# Body composition derangements in lung cancer patients treated with first‐line pembrolizumab: A multicentre observational study

**DOI:** 10.1002/jcsm.13568

**Published:** 2024-10-22

**Authors:** Ilaria Trestini, Lorenzo Belluomini, Alessandra Dodi, Marco Sposito, Alberto Caldart, Dzenete Kadrija, Luca Pasqualin, Silvia Teresa Riva, Ilaria Mariangela Scaglione, Daniela Tregnago, Alice Avancini, Jessica Insolda, Linda Confortini, Miriam Casali, Jessica Menis, Emanuele Vita, Marco Cintoni, Marco Todesco, Gianluca Milanese, Isabella Sperduti, Mirko D'Onofrio, Marco Infante, Marcello Tiseo, Maria Cristina Mele, Giampaolo Tortora, Michele Milella, Emilio Bria, Sara Pilotto

**Affiliations:** ^1^ Dietetic Service, Hospital Medical Direction University and Hospital Trust (AOUI) of Verona Verona Italy; ^2^ Department of Engineering for Innovation Medicine (DIMI), Section of Innovation Biomedicine ‐ Oncology Area University of Verona and University and Hospital Trust (AOUI) of Verona Verona Italy; ^3^ Medical Oncology Unit Hospital of Bolzano Bolzano Italy; ^4^ Department of Medical Oncology Santa Chiara Hospital Trento Italy; ^5^ Department of Medical Oncology IRCCS San Raffaele Scientific Institute Milan Italy; ^6^ Medical Oncology Unit Hospital ‘ASST di Lodi’ Lodi Italy; ^7^ Department of Traslational Medicine and Surgery, Medical Oncology Unit Università Cattolica del Sacro Cuore Rome Italy; ^8^ Department of Medical and Surgical Sciences, Department of Translational Medicine and Surgery, Clinical Nutrition Unit Fondazione Policlinico Universitario Agostino Gemelli IRCCS, Rome, Italy; Research and Training Center in Human Nutrition, Università Cattolica del Sacro Cuore Rome Italy; ^9^ Department of Radiology University of Verona and University and Hospital Trust (AOUI) of Verona Verona Italy; ^10^ Department of Medicine and Surgery (DiMeC), Section of Radiology, Unit of Surgical Sciences University of Parma Parma Italy; ^11^ Biostatistics Unit IRCCS Regina Elena National Cancer Institute Rome Italy; ^12^ Section of Diabetes & Metabolic Disorders, UniCamillus Saint Camillus International University of Health Sciences Rome Italy; ^13^ Department of Medicine and Surgery Parma, Italy; Medical Oncology Unit University Hospital of Parma University Hospital of Parma Parma Italy; ^14^ Università Cattolica del Sacro Cuore, Fondazione Policlinico Universitario Agostino Gemelli Rome Italy; ^15^ Ospedale Isola Tiberina ‐ Gemelli Isola Rome Italy

**Keywords:** Body composition, Immunotherapy, Intermuscular adipose tissue, Non‐small‐cell lung cancer, Sarcopenia

## Abstract

**Background:**

While immune checkpoint inhibitors (ICIs) are increasingly reshaping the therapeutic landscape of non‐small‐cell lung cancer (NSCLC), only a limited proportion of patients achieve a relevant and long‐lasting benefit with these treatments, calling for the identification of clinical and, ideally modifiable, predictors of efficacy. Body composition phenotypes may reflect aspects of patients' immunology and thereby their ability to respond to ICIs. This study aims to explore the possible association between pre‐treatment body composition phenotypes, tumour response, and clinical outcomes in patients receiving first‐line pembrolizumab monotherapy for advanced NSCLC.

**Methods:**

A retrospective review of consecutive patients with treatment‐naïve NSCLC and PD‐L1 expression ≥50% undergoing pembrolizumab at three academic institutions was performed. Pre‐treatment body composition parameters were measured at the third lumbar vertebra level by computed tomography, defined using pre‐established cut‐offs. Primary endpoint was objective response rate (ORR), secondary endpoints progression‐free survival and overall survival (PFS and OS), compared through the log‐rank test and the Cox proportional hazards model.

**Results:**

Data from 134 patients (93 males [69.4%] and 41 females [30.6%]) were collected. Median age was 69 years (range 36–85), with a median follow‐up of 12 months (range 1–131). The median body mass index (BMI) was 24.5 (IQR 21.5; 26.1) kg/m^2^. Overall, 59.0% and 51.5% of patients met established radiographic criteria for evidence of sarcopenia and myosteatosis, respectively, which occur across the BMI spectrum. Multivariate regression analysis, adjusted for co‐morbidities, revealed that sarcopenia (aOR 5.56, 95% CI. 2.46–12.6, *P* < 0.0001) and low intermuscular adipose tissue (IMAT) area (aOR 1.83, 95% CI. 1.22–2.83, *P* = 0.001) were associated with a lower rate of ORR (30.4% vs. 70.5%, *P* < 0.0001 and 30.7% vs. 73.2%, *P* < 0.0001, respectively). Moreover, both in univariate and multivariate analysis, adjusted for co‐morbidities, low performance status according to the Eastern Cooperative Oncology Group scale (ECOG PS), sarcopenia and low IMAT were significantly related to short PFS (ECOG PS: aHR 2.73, 95% CI 1.60–4.66, *P* < 0.0001; sarcopenia: aHR 2.24, 95% CI 1.37–3.67, *P* = 0.001; IMAT depot: aHR 2.26, 95% 1.40–3.63, *P* = 0.002) and OS (ECOG PS: aHR 3.44, 95% CI 1.96–6.01, *P* < 0.0001; sarcopenia: aHR 4.68, 95% CI 2.44–8.99, *P* < 0.0001; IMAT depot: aHR 3.18, 95% 1.72–5.88, *P* < 0.0001).

**Conclusions:**

Skeletal muscle abnormalities, apparently frequent in NSCLC, potentially represent intriguing predictive markers of response to ICIs and survival outcomes. Large prospective trials are needed to validate ICIs responders' clinical biomarkers.

## Introduction

Immune checkpoint inhibitors (ICIs), targeting programmed cell death protein 1 (PD‐1) and programmed death‐ligand 1 (PD‐L1), opened a novel landscape of treatment algorithms in advanced non‐small‐cell lung cancer (NSCLC).^1^

Given the aforementioned therapeutic advances, which broadened the treatment options, a proper selection of NSCLC patients is required from a clinical and pathological standpoint, to address the right therapy of choice. Nonetheless, although these agents are appealing options for medical oncologists treating patients with NSCLC due to their promise of durable responses, only a minority of patients experience durable clinical benefits from treatment with ICIs.^2^

Significant research efforts toward identifying optimal biomarkers that predict clinical outcomes after ICI treatment are currently underway. Various predictive biomarkers of immunotherapy such as PD‐L1 expression and tumour mutation burden have been reported.^3^ To date, PD‐L1 expression represent the only reliable biomarker, guiding, in clinical practice, the treatment choice. In this light, ICIs, both in combination with chemotherapy and in monotherapy, represent the upfront standard‐of‐care in patients with PD‐L1 negative or 1–49% and PD‐L1 ≥ 50%, respectively.^4^, ^5^, ^6^, ^7^, ^8^, ^9^ However, there is an unmet need for a well‐validated predictive risk‐scoring system for NSCLC patients starting treatment with ICIs. Moreover, identification of clinical, and ideally modifiable, predictors of ICI efficacy is a crucial goal in the immunotherapy era.

In this context, the relationship between nutritional status and homeostasis of the immune system and anticancer immunity could represent a recent fascinating area of research.^10^ Particularly, body composition phenotypes may reflect aspects of the patient's immunology and, thereby, his ability to promote a protective antitumor immune response.^11^ Despite increasing interest in this field and the nutritional derangements are common presentation hallmarks in patients affected by NSCLC,^12^ baseline nutritional assessment is usually under‐recognized in routine clinical practice.^13^, ^14^

To date, body composition can be evaluated via a range of different methods, including dual‐energy X‐ray absorptiometry (DXA) scan, bioelectrical impedance analysis (BIA), and computed tomography images (CT). DXA requires special equipment, and the accuracy of BIA is affected by dehydration, which is commonly observed in patients with advanced‐stage cancer. Of note, patients with cancer routinely undergo CT for cancer diagnosis, staging and assessment during therapy.^15^ It represents an easy modality to provide a careful assessment of body composition at specific and relevant time points throughout the entire course of patient's treatment, across different body weights, and hence body mass index (BMI) spectra.^16^

Given these perspectives, the herein reported multi‐center observational study aimed to (1) describe the pre‐treatment body composition profile of patients who received first‐line pembrolizumab monotherapy for advanced NSCLC; (2) examine the possible association between baseline body composition parameters and clinical outcomes in a homogeneous subgroup of patients who received first‐line pembrolizumab monotherapy for advanced NSCLC.

## Methods

### Study design

A multi‐center retrospective observational study was undertaken in consecutive patients with a diagnosis of advanced NSCLC with immunohistochemistry (IHC) PD‐L1 expression ≥50% who were treated with first‐line pembrolizumab monotherapy between August 2017 and August 2023. The recruiting centers were the following: (1) Medical Oncology of the University Hospital and Trust of Verona (Italy); (2) Medical Oncology of the Università Cattolica del Sacro Cuore, Fondazione Policlinico Universitario Agostino Gemelli, I.R.C.C.S., Roma (Italy); (3) Medical Oncology Unit of the University Hospital of Parma (Italy). The inclusion criteria were: age >18 years; confirmed histological diagnosis of advanced NSCLC; previous use of more than one infusion of pembrolizumab as front‐line therapy by medical choice within the framework of good clinical practice and in agreement with current guidelines; availability of pre‐treatment abdominal CT scans, performed before starting the immunotherapy (no more than 3 months earlier). Patients with degraded CT images insufficient to perform the analysis of body composition were excluded.

### Ethical approval

The study was approved by the local Ethical Committees (Prot. 2193CESC, 2022) and was conducted according to the ethical standards laid down in the 1964 Declaration of Helsinki and its later amendments. Clinical data were collected in a computerized database and anonymized before the analysis.

### Anthropometric measurements and body composition assessment

Weight and height obtained from the patient's chart were recorded by the hospital staff. Weight was measured with a medical balance beam scale and height was measured with a Harpenden stadiometer. BMI was calculated by dividing the weight in kilograms by the square of height in meters (kg/m^2^). Based on BMI values, patients were categorized as underweight (BMI less than 18.5 kg/m^2^), normal weight (BMI greater than or equal to 18.5 and 24.9 kg/m^2^), overweight (BMI greater than or equal to 25 to 29.9 kg/m^2^), class I obesity (BMI between 30 and 34.9 kg/m^2^), class II obesity (BMI between 35 and 39.9 kg/m^2^), and class III obesity (BMI greater than or equal to 40 kg/m^2^), based on the World Health Organization (WHO) criteria.^17^ We also investigated if there was a clinically significant weight loss, defined as at least a 5% reduction in weight within 6 months before disease presentation.^18^

Body composition was detected using CT image analysis, which was routinely performed among patients before starting immunotherapy, as it has previously been validated for use in the oncological setting.^15^ Cross‐sectional skeletal muscle and adipose tissue areas, in centimetres squared (cm^2^), from a single axial image at the third lumbar (L3) vertebrae are well correlated with whole‐body tissue volumes.^19^ The L3 images were analysed using a free‐form segmentation that closely approximated the Alberta protocol, a standard attenuation‐constrained segmentation protocol, via the built‐in tools provided by the SliceOmatic software version 5.0 (TomoVision, 3,280 Chemin Milletta, Magog, J1X 0R4, Canada), distinguishing muscle from visceral, subcutaneous and intermuscular adipose tissues using anatomic knowledge and tissue‐specific Hounsfield unit (HU) ranges. Skeletal muscle area (SMA) was identified between −29 and +150 HU, visceral adipose tissue (VAT) was identified between −150 and −50 HU, subcutaneous adipose tissue (SAT) and intermuscular adipose tissue (IMAT) between −190 and −30 HU, as it has previously been published.^20^ The muscles in the L3 region include the psoas, erector spinae, quadratus lumborum, transversus abdominus, external and internal obliques, and rectus abdominus. SMA was normalized by dividing the body surface area and defined as skeletal muscle index (SMI) (cm^2^/m^2^). This process was necessary to accurately determine the muscle mass volume, which was greatly affected by body shape. We also converted the other areas to indices [Intermuscular Fat Index (IMFI), Visceral Fat Index (VFI), and Subcutaneous Fat Index (SFI)], by dividing them by height in meters squared.

Sex‐specific, BMI‐stratified SMI cut‐off values published by Martin et al. were applied for classifying patients with sarcopenia.^16^ Sarcopenia was defined as SMI of <43 cm^2^/m^2^ for men with BMI < 25 kg/m^2^, SMI < 53 cm^2^/m^2^ for men with BMI ≥ 25 kg/m^2^, and SMI < 41 cm^2^/m^2^ for women.

Mean muscle attenuation, as expressed as the mean HU, was also evaluated. It indirectly measures fat infiltration in muscle and has been extensively studied as a correlate of muscle radiodensity, a measure of muscle quality.^21^ The sex‐specific cut‐off values for myosteatosis were 28.6 HU for female patients and 38.5 HU for male patients.^22^ Figure S1 provides an example of comparison of axial CT images between two male patients with the same BMI.

### Data collection and end‐points

We retrospectively collected data on patient demographics, Eastern Cooperative Oncology Group (ECOG) performance status (PS), smoking status, histological subtype, stage, PD‐L1 status, molecular status, and co‐morbidities.

The primary endpoint of this study was the objective response rate (ORR). Response assessment to treatment was conducted, retrospectively, according to Response Evaluation Criteria in Solid Tumours (RECIST) 1.1 criteria.^23^ Patients were categorized according to their best response to ICIs as having complete response (CR), partial response (PR), stable disease (SD), and progressive disease (PD); ORR was defined as the percentage of individuals who achieved CR or PR as the best response to treatment.

Secondary endpoints included progression‐free survival and overall survival (PFS, OS). PFS was calculated from the initiation of ICIs until the date of disease progression or death. OS was calculated from the beginning of ICIs until the date of death from any cause. The follow‐up date was ended on 9 August 2023.

### Statistical analysis

Statistical analysis was performed using SPSS version 18 (SPSS, Chicago, IL, UA) and MedCalc Version 14.2.1 (MedCalc Software Ostend, Belgium) licensed statistical programs. The different variables contained in the study were analysed with descriptive statistics. Continuous data were expressed as medians with interquartile ranges (IQR). Categorical variables were expressed as numbers and percentages. Continuous variables were analysed using the Mann–Whitney *U* test, while categorical variables were analysed using Fisher's exact test. Chi‐squared test was used to compare the treatment response between the sarcopenia and non‐sarcopenia groups, while the student t‐test or Mann–Whitney *U* test were used to test any difference in body composition variables between patients with objective response and those without OR.

The variables included in the univariate analysis for ORR, PFS, and OS were: age at diagnosis, gender, ECOG performance status, smoking status, weight loss, pre‐treatment significant weight loss (considered clinically relevant if more than 5% of pre‐diagnosis weight), BMI, SMA, SMI, sarcopenia, sarcopenic obesity, SAT, SFI, VAT, VFI, IMAT, IMFI, SMR, and myosteatosis. Variables significantly associated with ORR, PFS, and OS at univariate analysis were entered into the Cox multivariate analysis to assess their independent character.

The Logistic Regression model and the Cox Regression model with proportional risk with clinical‐pathological and body composition features were developed using gradual regression (forward selection, enter limit and remove the limit, *P* = 0.10, and *P* = 0.15, respectively), to identify independent predictive and prognostic, respectively. Odds ratio (OR) and hazard ratio (HR) with 95% confidence intervals (95% CI) were estimated for each variable according to the logistic and Cox regression model for the ORR and PFS as well as OS, respectively. Specifically, to test if the association between body composition parameters and clinical outcomes was independent from the presence of concurrent co‐morbidities, they were entered in the models, obtaining adjusted OR (aOR) and adjusted HR (aHR).

Survival curves were plotted using the Kaplan–Meier method and compared between groups using the log‐rank test.

The receiver operating characteristic (ROC) curves of selected parameters searching for the best cut‐offs for the population were calculated. The area under the curve (AUC) ≥ 0.5 was considered to have a diagnostic value, the larger the area, the larger the value. All *P*‐values were two‐sided, and a *P*‐value < 0.05 was considered statistically significant.

## Results

### Patients' clinical features

Based on the inclusion criteria, 134 patients were included in the study. Patients' characteristics are depicted in Table 1. Ninety‐three (69.4%) patients were males, and the median age was 69 years (range: 36–85 years). Approximately 52.2% of patients had any time history of smoking, and 38.8% were active smokers at the time of treatment initiation. The baseline ECOG PS distribution was as follows: 0 (39.6%), 1 (43.3%), 2 (15.7%), and 3 (1.5%). The most represented tumour histology was adenocarcinoma (73.1%). More than a third of patients (41.0%) was submitted to radiotherapy during immunotherapy. During the observation period, grade 3 or 4 immune‐related adverse events, defined as severe or potentially life‐threatening toxicities requiring intervention and hospitalization, occurred in 14 patients (10.4%), including 7 (50%) cases of colitis, 5 (35.7%) cases of dermatological adverse events, 3 (21.4%) cases of pneumonitis, and 1 (7.1%) case of hepatic adverse event (increased levels of alanine aminotransferase and aspartate transaminase).

**Table 1:** Baseline patients' clinical characteristics for the whole population

Patients' characteristics	Number of patients, *n* = 134 (%)
Age at diagnosis (years)	
Median (range)	69 (36–85)
Gender	
Female	41 (30.6%)
Male	93 (69.4%)
ECOG performance status	
0–1	111 (82.8%)
≥2	23 (17.2%)
Smoking status	
Current	52 (38.8%)
Former	70 (52.2%)
Never	5 (3.7%)
Not evaluated	7 (5.2%)
Pre‐existent co‐morbidities	
Cardiovascular disease	70 (52.2%)
Diabetes mellitus	38 (28.4%)
COPD	21 (15.7%)
Chronic kidney disease	8 (6.0%)
Gastrointestinal disorders	6 (4.5%)
Infections	6 (4.5%)
Nervous system disease	3 (2.4%)
Patients with 2 or more co‐morbidities	43 (32.1%)
Tumour histology	
Adenocarcinoma	98 (73.1%)
Squamous cell carcinoma	25 (18.7%)
Other	11 (8.2%)
Disease stage	
III	24 (17.9%)
IV	110 (82.1%)
Metastatic sites	
Lung and pleura	65 (48.5%)
Lymph nodes	81 (60.5%)
Bone	33 (24.6%)
Liver	11 (8.2%)
Brain	30 (22.4%)
Other	26 (19.4%)
Number of metastatic sites	
<3	69 (51.5%)
≥3	47 (35.1%)
PD‐L1 values	
Median (IQR)	70 (50–90)
Molecular status	
EGFR (121 with available data)	0 (0%)
ALK (127 with available data)	0 (0%)
ROS1 (133 with available data)	0 (0%)
KRAS (44 with available data)	13 (9.7%)
Radiotherapy	
Yes	55 (41.0%)
No	79 (58.9%)

ALK, anaplastic lymphoma kinase; COPD, chronic obstructive pulmonary disease; ECOG, Eastern Cooperative Oncology Group; EGFR, epidermal growth factor receptor; KRAS, Kirsten Rat Sarcoma; PD‐L1, programmed death ligand 1.

### Baseline anthropometric and body composition characteristics

Baseline anthropometric and body composition features of the patient population, according to gender, are shown in Table 2. A history of weight loss during the 6 months before starting immunotherapy was common, with a median loss of 4.1 (IQR 0; 5.5) %. Overall, 24.6% reported a clinically significant weight loss. However, because of the generally heavy body weights, many patients remained overweight despite considerable weight loss. Analysis of BMI showed a median BMI of 24.5 (IQR 21.6; 26.1) kg/m^2^, with 32.8% and 4.5% of patients overweight and obese, respectively, and with only 6.0% who presented as underweight. In terms of muscle and fat composition of these patients, the median values for SMA, SMI, SAT, SFI, VAT, VFI, IMAT, IMFI, and SMR for females and males, respectively, are shown in Table 2. Skeletal muscle abnormalities were frequently present in our cohort of patients, in all BMI categories. The overall prevalence of sarcopenia, according to the Martin criteria^16^ was 59.0%. Among these patients, 3 (3.8%) were categorized as obese, 27 (34.2%) as overweight, 46 (58.2%) as normal weight, and 3 (3.8%) as underweight. Our data did not show sex differences in SMA and SMI.

**Table 2:** Baseline anthropometric and body composition characteristics, according to gender

Variables	Overall patients *n* = 134 (%)	Female (*n* = 41)	Male (*n* = 93)	*P*‐value
Weight (kg)				
Median (IQR)	70 (60; 70)	58 (50.5; 58)	73 (65.5; 83)	0.097
Weight loss (%)				
Median (IQR)	4.1 (0; 5.5)	6.4 (1.2; 11.1)	2.3 (−1.6;4.9)	0.092
BMI (kg/m^2^)				
Median (IQR)	24.5 (21.6; 26.1)	23.4 (20.1; 23.1)	24.6 (22.9;26.0)	0.083
Underweight	8 (6.0%)	5 (12.2%)	3 (3.2%)	
Normal weight	76 (56.7%)	22 (53.6%)	54 (58.1%)	
Overweight	44 (32.8%)	12 (29.3%)	32 (34.4%)	
Obese class I	4 (3.0%)	1 (2.4%)	3 (3.2%)	
Obese class II	2 (1.5%)	1 (2.4%)	1 (1.1%)	
SMA (cm^2^)				
Median (IQR)	125.7 (106.3; 145.1)	100.8 (84.3; 99.3)	138.3 (121.5; 155.8)	0.33
SMI (cm^2^/m^2^)				
Median (IQR)	42.8 (37.6; 50.1)	37.5 (32.6; 37.3)	45.5 (41.6; 52.0)	0.43
Sarcopenia	79 (59.0%)	28 (68.3%)	51 (54.8%)	0.78
Sarcopenic obesity	3 (2.2%)	1 (2.4%)	2 (1.02%)	0.071
SAT (cm^2^)				
Median (IQR)	141.3 (97.2; 183.6)	155.4 (94.8; 151.5)	140.6 (101.1; 178.7)	0.210
SFI (cm^2^/m^2^)				
Median (IQR)	49.0 (35.9; 67.0)	54.2 (36.1; 53.5)	46.3 (35.0; 58.7)	0.33
VAT (cm^2^)				
Median (IQR)	113.0 (54.6; 196.3)	89.4 (29.1; 89.3)	143.3 (75.7; 227.1)	0.095
VFI (cm^2^/m^2^)				
Median (IQR)	41.2 (18.8; 66.7)	32.9 (11.7; 32.0)	52.8 (25.1; 71.4)	0.081
IMAT (cm^2^)				
Median (IQR)	13.1 (9.3; 25.3)	12.6 (9.3; 12.4)	13.9 (9.4; 24.8)	0.931
IMFI (cm^2^/m^2^)				
Median (IQR)	4.7 (3.4; 8.4)	4.7 (3.6; 4.5)	4.7 (3.0; 7.8)	0.863
SMR (HU)				0.125
Median (IQR)	32.1 (25.8; 38.6)	30.6 (26.9; 30.6)	32.5 (25.7; 39.9)	
Myosteatosis	69 (51.5%)	14 (20.3%)	55 (79.7%)	**0.001**

BMI, body mass index; HU, Hounsfield units; IMAT, intermuscular adipose tissue; IMFI, intermuscular fat index; SAT, subcutaneous adipose tissue; SFI, subcutaneous fat index; SMA, skeletal muscle area; SMI, skeletal muscle index; SMR, skeletal muscle radiodensity; VAT, visceral adipose tissue; VFI, visceral fat index.

The prevalence of myosteatosis was 51.5%, of which 6 patients (8.7%) were categorized as obese, 32 (46.4%) as overweight, 29 (42.0%) as normal weight, and only 2 (2.9%) as underweight. A very high proportion of males met the criteria for myosteatosis (79.7% of males) as compared with females (20.3% of females) (*P* < 0.001). Patients with myosteatosis, as compared with patients without myosteatosis, were older (*P* < 0.0001) and had a higher BMI (*P* = 0.006).

### Correlation between body mass index and body composition

BMI was significantly and positively correlated with SMA (*r* = 0.448, *P* < 0.0001) and SMI (*r* = 0.439, *P* < 0.0001), while significantly and negatively correlated with SMR (*r* = −0.252, *P* = 0.006). A significant positive correlation was also found between BMI, and SAT (*r* = 0.699, *P* < 0.0001), SFI (*r* = 0.650, *P* < 0.0001), VAT (*r* = 0.598, *P* < 0.0001), VFI (*r* = 0.595, *P* < 0.0001) as well as BMI and IMAT (*r* = 0.283, *P* = 0.002) and IMFI (*r* = 0.251, *P* = 0.007).

### Association between body composition and clinical outcomes

Median follow‐up time was 12 months (range 1–131). Data regarding response to ICIs and patient survival (median PFS, median OS) are shown in Table S1.

#### Primary endpoint: Treatment response

Best response achieved included a CR in 6 (4.5%) patients and a partial response (PR) in 57 (42.5%) patients, resulting in an ORR value of 47.0%.

**Figure 1:**
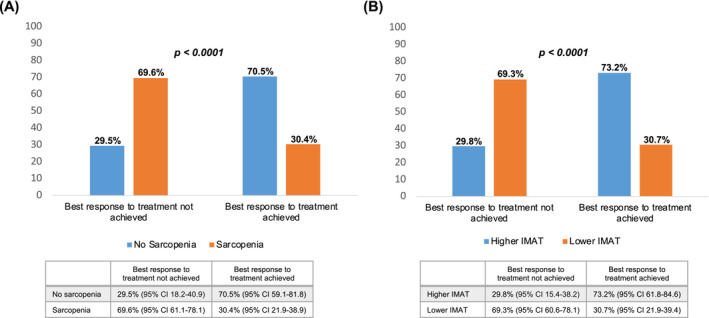
Correlation between objective response rate (ORR), sarcopenia (A), and IMAT (B).

The associations between body composition parameters and ORR are reported in Table 3. In univariate analysis, SMA, SMI, sarcopenia, IMAT, IMFI, SMR, and myosteatosis were significantly related to ORR. Multivariate regression analysis revealed that sarcopenia (aOR 5.56, 95% CI. 2.46–12.6, *P* < 0.0001) and low IMAT area (aOR 1.83, 95% CI. 1.22–2.83, *P* = 0.001) were associated with lower ORR. The ORR in patients without sarcopenia was significantly higher compared with the sarcopenic patients (70.5% vs. 30.4%, *P* < 0.0001) (Figure 1A). Patients with pre‐treatment higher IMAT accumulation had significantly better ORR compared with patients with lower IMAT area (73.2% vs. 30.7%, *P* < 0.0001) (Figure 1B).

**Table 3:** Univariate and multivariate logistic regression analysis for ORR

Characteristics	Variables	OR	95% CI	*P*‐value	OR	95% CI	*P*‐value
Age (years)	Analysed as a continuous variable	1.02	0.98–1.06	0.263			
Gender	Male vs. female	1.38	0.66–2.90	0.323			
ECOG PS	2 vs. 1–2	2.33	0.64–8.49	0.199			
Smoking status	No or former vs. Yes	3.74	0.41–34.49	0.244			
Weight loss (%)	Analysed as a continuous variable	0.97	0.92–1.02	0.235			
Weight loss (%)	≥5% vs. <5%	0.97	0.92–1.02	0.068			
BMI (kg/m^2^)	Analysed as a continuous variable	0.99	0.90–1.01	0.892			
BMI (kg/m^2^)	≤25 vs. >25	1.06	0.51–2.18	0.880			
SMA (cm^2^)	Analysed as a continuous variable	1.02	1.00–1.03	0.012			
SMI (cm^2^/m^2^)	Analysed as a continuous variable	1.06	1.02–1.11	0.008			
Sarcopenia	Yes vs. no	5.46^a^	2.44–12.2	<0.0001	5.56^a^	2.46–12.6	<0.0001
Sarcopenic obesity	Yes vs. no	1.75	0.16–19.8	0.651			
SAT (cm^2^)	Analysed as a continuous variable	0.99	0.99–1.03	0.474			
SFI (cm^2^/m^2^)	Analysed as a continuous variable	0.99	0.99–1.01	0.365			
VAT (cm^2^)	Analysed as a continuous variable	1.0	0.99–1.05	0.563			
VFI (cm^2^/m^2^)	Analysed as a continuous variable	1.0	0.99–1.02	0.583			
IMAT (cm^2^)	Analysed as a continuous variable	1.08^a^	1.03–1.12	<0.0001	1.83^a^	1.22–2.83	0.001
IMFI (cm^2^/m^2^)	Analysed as a continuous variable	1.21	1.08–1.34	0.001			
SMR (HU)	Analysed as a continuous variable	0.95	0.91–0.99	0.024			
Myosteatosis	Yes vs. no	3.06	1.39–6.72	0.005			

BMI, body mass index; ECOG, Eastern Cooperative Oncology Group; HU, Hounsfield units; IMAT, intermuscular adipose tissue; IMFI, intermuscular fat index; OR, odds ratio; SAT, subcutaneous adipose tissue; SFI, subcutaneous fat index; SMA, skeletal muscle area; SMI, skeletal muscle index; SMR, skeletal muscle radiodensity; VAT, visceral adipose tissue; VFI, visceral fat index.

^a^ Adjusted for co‐morbidities.

#### Secondary endpoint: Progression‐free survival

In all patients, the median PFS was 7.4 months (95% CI 5.6–9.2). One‐ and two‐year PFS rates were 36.5% and 25.2%, respectively. Table 4 summarizes the associations between body composition parameters and PFS. In univariable Cox regression analysis, ECOG PS, SMA, SMI, sarcopenia, IMAT, IMFI, and myosteatosis were significantly associated with PFS.

**Table 4:** Univariate and multivariate analysis using the cox regression method investigating the hazard ratios of baseline patient's characteristics on PFS

Characteristics	Variables	HR	95% CI	*P*‐value	HR	95% CI	*P*‐value
Age (years)	Analysed as a continuous variable	0.99	0.97–1.01	0.526			
Gender	Male vs. Female	1.09	0.72–1.67	0.683			
ECOG PS	2 vs. 1–2	3.91^a^	2.41–6.34	<0.0001	2.73^a^	1.60‐4.66	<0.0001
Smoking status	No or former vs. yes	3.23	1.28–8.14	0.013			
Weight loss (%)	Analysed as a continuous variable	1.01	0.98–1.03	0.723			
Weight loss (%)	≥5% vs. <5%	1.51	0.97–2.36	0.068			
BMI (kg/m^2^)	Analysed as a continuous variable	1.02	0.96–1.01	0.509			
BMI (kg/m^2^)	≤25 vs. >25	1.0	0.99–1.04	0.892			
SMA (cm^2^)	Analysed as a continuous variable	0.99	0.98–0.99	0.021			
SMI (cm^2^/m^2^)	Analysed as a continuous variable	0.96	0.94–0.99	0.003			
Sarcopenia	Yes vs. no	2.96^a^	1.89–4.64	<0.0001	2.24^a^	1.37‐3.67	0.001
Sarcopenic obesity	Yes vs. no	3.02	0.94–9.7	0.064			
SAT (cm^2^)	Analysed as a continuous variable	1.0	0.99–1.04	0.369			
SFI (cm^2^/m^2^)	Analysed as a continuous variable	1.0	0.99–1.03	0.345			
VAT (cm^2^)	Analysed as a continuous variable	1.0	0.99–1.03	0.810			
VFI (cm^2^/m^2^)	Analysed as a continuous variable	1.0	0.99–1.08	0.860			
IMAT (cm^2^)	Analysed as a continuous variable	0.97^a^	0.95–0.99	0.002	2.26^a^	1.40–3.63	0.002
IMFI (cm^2^/m^2^)	Analysed as a continuous variable	0.92	0.87–0.97	0.003			
SMR (HU)	Analysed as a continuous variable	1.02	0.99–1.04	0.127			
Myosteatosis	Yes vs. no	1.48	0.97–2.23	0.063			

BMI, body mass index; ECOG, Eastern Cooperative Oncology Group; HU, Hounsfield units; IMAT, intermuscular adipose tissue; IMFI, intermuscular fat index; SAT, subcutaneous adipose tissue; SFI, subcutaneous fat index; SMA, skeletal muscle area; SMI, skeletal muscle index; SMR, skeletal muscle radiodensity; VAT, visceral adipose tissue; VFI, visceral fat index.

^a^ Adjusted for co‐morbidities.

**Figure 2:**
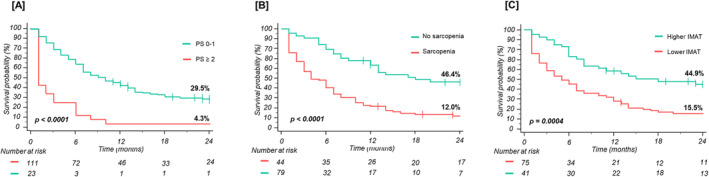
Progression‐free survival (PFS) curves according to ECOG PS (A), sarcopenia (B), and IMAT (C).

In multivariable analysis, ECOG PS (aHR 2.73, 95% CI 1.60–4.66, *P* < 0.0001), sarcopenia (aHR 2.24, 95% CI 1.37–3.67, *P* = 0.001), and IMAT area (aHR 2.26, 95% 1.40–3.63, *P* = 0.002) remained independent predictors of PFS. In particular, the Kaplan–Meier analysis revealed that, compared with patients with an ECOG PS of 0 or 1, patients with an ECOG PS ≥ 2 had a significantly lower PFS (2‐year PFS rates 29.5% vs. 4.3% *P* < 0.0001; Figure 2A). Patients with sarcopenia had a significantly shorter PFS than those without sarcopenia (2‐year PFS rates 12% vs. 46.4%, *P* < 0.0001; Figure 2B). The stratified analysis based on the stage confirmed the association between sarcopenia and PFS (*P* < 0.0001) (Figure S2B).

A change point method based on the log‐rank test was applied to the IMAT values to estimate the most appropriate cut‐off able to split patients into groups with different PFS probabilities: the optimal cut‐off was 19.94 cm^2^ (AUC 0.775 with a standard error of 0.05). Patients with pre‐treatment lower IMAT accumulation had significantly poorer PFS compared with patients with higher IMAT area (2‐year PFS rates 15.5% vs. 44.9%, *P* = 0.0004) (Figure 2C).

#### Secondary endpoint: Overall survival

**Figure 3:**
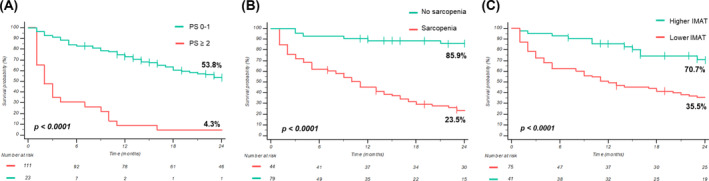
Overall survival (OS) curves according to ECOG PS (A), sarcopenia (B), and IMAT (C).

Of the 134 patients, 88 (65.7%) had died before the cut‐off date. The median OS from immunotherapy treatment initiation was 19.3 months (95% CI 12.7–26) in all patients. One‐ and two‐year OS rates were 61.8% and 44.2%, respectively. Investigating nutritional parameters in relation to OS (Table 5), pre‐treatment significant weight loss, as well as weight loss analysed as a continuous variable, SMA, SMI, sarcopenia, IMAT, IMFI, and myosteatosis were significantly correlated to OS. In multivariate analysis for OS, ECOG PS (aHR 3.44, 95% CI 1.96–6.01, *P* < 0.0001), sarcopenia (aHR 4.68, 95% CI 2.44–8.99, *P* < 0.0001), and IMAT depot (aHR 3.18, 95% 1.72–5.88, *P* < 0.0001) maintained their independent statistical significance. Particularly, patients with an ECOG PS of ≥2 had a significantly worse OS compared with patients with an ECOG PS of 0 or 1 (2‐year PFS rates 4.3% vs. 53.8% *P* < 0.0001; Figure 3A).

**Table 5:** Univariate and multivariate analysis using the cox regression method investigating the hazard ratios of baseline patient's characteristics on OS

Characteristics	Variables	HR	95% CI	*P*‐value	HR	95% CI	*P*‐value
Age (years)	Analysed as a continuous variable	0.99	0.97–1.02	0.767			
Gender	Male vs. female	1.03	0.65–1.63	0.891			
ECOG PS	2 vs. 0–1	6.11^a^	3.64–10.3	<0.0001	3.44^a^	1.96‐6.01	<0.0001
Smoking status	No or former vs. yes	1.72	0.69–4.26	0.241			
Weight loss (%)	Analysed as a continuous variable	1.03	1.01–1.05	0.011			
Weight loss (%)	≥5% vs. <5%	2.53	1.56–4.01	<0.0001			
BMI (kg/m^2^)	Analysed as a continuous variable	0.99	0.93–1.05	0.736			
BMI (kg/m^2^)	≤25 vs. >25	0.97	0.99–1.02	0.583			
SMA (cm^2^)	Analysed as a continuous variable	0.99	0.98–0.99	<0.0001			
SMI (cm^2^/m^2^)	Analysed as a continuous variable	0.94	0.92–0.97	<0.0001			
Sarcopenia	Yes vs. no	7.06^a^	3.79–13.2	<0.0001	4.68^a^	2.44‐8.99	<0.0001
Sarcopenic obesity	Yes vs. no	2.32	0.73–7.4	0.154			
SAT (cm^2^)	Analysed as a continuous variable	1.0	0.99–1.01	0.906			
SFI (cm^2^/m^2^)	Analysed as a continuous variable	1.0	0.99–1.01	0.936			
VAT (cm^2^)	Analysed as a continuous variable	1.0	0.99–1.03	0.894			
VFI (cm^2^/m^2^)	Analysed as a continuous variable	1.0	0.99–1.01	0.863			
IMAT (cm^2^)	Analysed as a continuous variable	0.95^a^	0.93–0.98	<0.0001	3.18^a^	1.72–5.88	<0.0001
IMFI (cm^2^/m^2^)	Analysed as a continuous variable	0.88	0.82–0.94	<0.0001			
SMR (HU)	Analysed as a continuous variable	1.01	0.98–1.01	0.432			
Myosteatosis	Yes vs. no	1.55	0.99–2.44	0.05			

BMI, body mass index; ECOG, Eastern Cooperative Oncology Group; HU, Hounsfield units; IMAT, intermuscular adipose tissue; IMFI, intermuscular fat index; SAT, subcutaneous adipose tissue; SFI, subcutaneous fat index; SMA, skeletal muscle area; SMI, skeletal muscle index; SMR, skeletal muscle radiodensity; VAT, visceral adipose tissue; VFI, visceral fat index.

^**a**^ Adjusted for co‐morbidities.

Patients with sarcopenia had a significantly shorter OS than those without sarcopenia (2‐year OS rates 85.9% vs. 23.5% *P* < 0.0001; Figure 3B), regardless of NSCLC stage (*P* < 0.0001; Figure S3B).

Patients with pre‐treatment higher IMAT depot had significantly longer OS compared with patients with lower IMAT (2‐year OS rates 70.7% vs. 35.5%, *P* < 0.0001, Figure 3C).

## Discussion

This study showed that pre‐treatment skeletal muscle wasting is a prominent feature in NSCLC patients. Most importantly, muscle abnormalities have been found to have a detrimental impact on ORR and have been associated with poor survival outcomes in patients with NSCLC treated with first‐line pembrolizumab monotherapy. Particularly, patients with sarcopenia had a significantly lower ORR than those without sarcopenia. Moreover, sarcopenia has been shown to be a poor predictive factor of PFS as well as of OS, both in univariate analysis and in multivariate analysis.

Our results add to the recent evidence that sarcopenia is associated with poor survival outcomes in the context of ICIs treatment for NSCLC^24^, ^25^ and other solid tumours.^26^ Of note, in our study, an early drop‐off of sarcopenic patients after starting ICI is evident, which mirrors the curve for ECOG PS. Although the precise reasons for this finding are not yet elucidated, previous research has shown a similar trend, suggesting that impaired treatment tolerability and efficacy in sarcopenic patients might be a potential cause of the early drop‐off.^27^ Among the possible explanations regarding the worse outcomes observed in patients with muscle wasting, detrimental effects on the immune system mediated by chronic inflammation, which lead to the capability of tumour cells to escape antitumor immune cell responses (immune escape), seem to play a key role.^10^ Muscle depletion, with impaired myokine signalling, is associated with decreased production and secretion of IL‐15 by skeletal muscle cells, along with high levels of IL‐6 and TGF‐β. The latter phenomenon can lead to the suppression of natural killer cells by mTOR inhibition, leading to their dysfunction and inability to effectively eliminate malignant cells, and contribute to impaired maintenance, proliferation, and survival of T‐cells, including CD8 + T‐cells, which are considered potential targets for PD‐1/PD‐L1 inhibitors.^28^ Muscle wasting is a phenotypic criterion of malnutrition and a prominent body composition phenotype in lung cancer patients.^29^ It reflects increased protein degradation, reduced protein synthesis, or a relative imbalance of the two processes, due to a complex interplay among cytokines, hormones, and other humoral factors, change in energy and substrate metabolism, and reduction in nutrient intake or availability, as well as in physical activity.^30^

In our cohort, almost half of NSCLC patients were found to be sarcopenic, according to the Martin criteria,^16^ in line with earlier reports on lung cancer.^31^, ^32^ Nevertheless, more than a quarter of patients were overweight or obese. Similarly, a study by Baracos et al., analysing data from a prospective cohort of NSCLC patients, reported that at presentation nearly half of the patients were overweight or obese, and among those classified as overweight more than half met the criteria for muscle depletion.^31^

Besides muscular depletion, skeletal muscle derangement is also characterized by an increased proportion of intermuscular fat, which is a marker of muscle quality deterioration.^33^ Quantification of IMAT area can be assessed by CT using specific software, through which adipose tissue can be clearly distinguished and digitally separated from skeletal muscle and other adipose tissue depots.^34^

In our study, 51.5% of patients met established radiographic criteria for evidence of myosteatosis, according to Martin et al.^16^ Interestingly, our study found that IMAT content was associated with ORR at multivariate analysis and that a high IMAT depot is associated with longer PFS, and OS. Unfortunately, only limited data are available on this compelling interest topic. Minami et al., in a retrospective analysis of 74 NSCLC patients who had received PD‐1/PD‐L1 inhibitor therapy, observed the absence of impact of IMAT on PFS.^35^

A retrospective study by Nishioka et al., among NSCLC patients treated with ICIs, reported that high muscle quality, defined using skeletal muscle density, correlates with higher ORR and longer PFS.^36^

Interestingly, consistent with the results of our study, a recent Japanese retrospective study conducted among patients with advanced gastric cancer treated with ICIs reported that patients with a high IMAT had significantly better survival outcomes.^37^

The IMAT values become greater with increasing BMI, as confirmed by the results of our study, and IMAT accumulation is positively associated with total body fat.^34^ Adipose tissue is a metabolically dynamic organ that is the primary site of energy storage and an endocrine organ capable of synthesizing several biologically active compounds that regulate metabolic homeostasis.^38^ The IMAT depot may be capable of secreting cytokines known to influence tissue inflammation and insulin sensitivity.^39^ A study of adipose tissue epigenetics in pigs found that IMAT has a methylation pattern like VAT, with increased expression of interleukin (IL)‐6 and tumour necrosis factor‐alpha (TNF‐α) compared with subcutaneous adipose depot.^40^ Additionally, a recent study suggests that IMAT is a unique adipose tissue depot with an immunogenic and inflammatory secretome. It seems to secrete statistically significantly higher levels of inflammatory cytokines and chemokines than SAT and VAT areas.^41^ Recent data showed an unexpected inverse association between adiposity and better outcomes in patients treated with ICIs, called the ‘obesity paradox’, both in preclinical models and in cancer patients.^10^ Mechanisms of this effect are unclear, although obesity may alter key inflammatory cytokines and promote a chronic low‐grade inflammation that modifies tumour‐infiltrating lymphocytes and tumour‐associated macrophage populations.^42^ Particularly, Wang et al. showed that in mice models, increased leptin concentration can lead to upregulation of PD‐1 receptors on T‐cells. That, in turn, can lead to an enhanced response to ICIs and immune cells mediated tumour regression.^43^

However, these body composition phenotypes are often hidden in clinical practice,^13^ which may be neglected using ‘standard’ anthropometric measurements such as weight and BMI. Early assessment and monitoring of body composition should be routinely carried out in lung cancer patients, despite normal or heavy body weight, since it could be relevant in the clinical decision‐making process before starting immunotherapy and for effective patient selection and stratification.

This study has several limitations, including the retrospective nature of the studies which is prone to sampling error. Of note, the OS results are potentially affected by a selection bias due to the presence of a limited percentage of patients (20%) needed to be alive at the moment of enrolment. This limitation derived from an ethical committee recommendation from one centre involved in the study. Therefore, we choose ORR as the primary endpoint, as it could represent a more reliable outcome measure, going over the prognostic impact of PFS and OS.

Moreover, patients were excluded from our analysis if an abdominal CT scan was not performed at baseline, which may lead to selection bias. This analysis, such as most research in cancer patients, used a low SMI as a marker of sarcopenia, while muscle strength and function have not been assessed. We could not study the body composition phenotype of ‘sarcopenic obesity’ specifically because we had only six patients with BMI ≥ 30 Kg/m^2^. This condition combines low muscle mass and high‐fat mass and seems to be associated in the literature with worst outcomes^44^ and with higher toxicity related to ICI treatment.^45^

Thus, the dynamic changes in body composition patterns observed during ICI treatment and even before the treatment initiation, which can further affect clinical outcomes, are neglected. Another limitation is related to the lack of information regarding concomitant therapies at the moment of disease diagnosis (prior to ICIs initiation) that might have influenced body composition (i.e., corticosteroids). Moreover, this cohort of patients (PD‐L1 ≥ 50%) was treated exclusively with ICI monotherapy (according to the current ICIs approval in Italy), thus excluding patients with PDL1 < 50%, treated with chemoimmunotherapy.

Finally, this study did not include a control cohort that did not receive immunotherapy, and therefore, it was difficult to make accurate assessments or conclusions regarding predictive roles. Therefore, these findings should be regarded as hypothesis‐generating for future studies.

However, this is a multicentre study among a relatively uniform group of patients in terms of diagnosis (advanced NSCLC) and treatment (pembrolizumab monotherapy).

## Conclusions

Skeletal muscle derangements were prevalent in patients affected by advanced NSCLC. Interestingly, sarcopenia has been found to negatively impact ORR, PFS, and OS in patients with advanced NSCLC treated with mono‐immunotherapy. In contrast and somewhat unexpected, higher IMAT depot was associated with higher ORR and superior PFS and OS. In this light, exploring interventional strategies (such as tailored nutritional counselling, personalized exercise) aimed at optimizing body composition toward a more immune‐responsive phenotype is extremely intriguing for promoting ICIs efficacy and potentially reversing resistance in a proportion of NSCLC patients.

## Funding

This research did not receive any specific grant from funding agencies in the public, commercial, or not‐for‐profit sectors.

## Conflict of interest

L.B. received speakers' fees from Astra‐Zeneca, MSD, Roche, Takeda and travel fees from Takeda and Sanofi. M.T. received speakers' and consultants' fee from Astra‐Zeneca, Pfizer, Eli‐Lilly, BMS, Novartis, Roche, MSD, Boehringer Ingelheim, Otsuka, Takeda, Pierre Fabre, Amgen, Merck, Sanofi, Janssen, and Daiichi Sankyo. M.T. received institutional research grants from Astra‐Zeneca and Boehringer Ingelheim. E.B. has received grants or contracts from Astra‐Zeneca, Roche and honoraria for lectures from Merck‐Sharp & Dome, Astra‐Zeneca, Pfizer, Eli‐Lilly, Bristol‐Myers Squibb, Novartis, Takeda, and Roche and has been a member of Data Safety Monitoring Board or Advisory Board of Merck‐Sharp & Dome, Pfizer, Novartis, Bristol‐Myers Squibb, Astra‐Zeneca, and Roche. S.P. received honoraria or speakers' fees from Astra‐Zeneca, Eli‐Lilly, BMS, MSD, Takeda, Amgen, Novartis, and Roche. The rest of the authors declare no conflict of interest.

## Supporting information


**Figure S1.** Comparison of axial CT images between two male patients with the same BMI. Legend Supplementary Data 4: In red, lumbar skeletal muscle area (SMA) (cm^2^); in yellow, visceral adipose tissue area (VAT) (cm^2^); in teal: subcutaneous adipose tissue area (SAT) (cm^2^); in green, intermuscular adipose tissue area (IMAT) (cm^2^). Both patients had a BMI of 26.2 kg/m^2^. According to the established skeletal muscle index (SMI) cut‐off values, the first patient (left panel) reported sarcopenia, with a SMI value of 48 (<53) cm^2^/m^2^, and myosteatosis with a skeletal muscle radiodensity (SMR) of 32.1 (<33) HU. The second patient (right panel) did not show sarcopenia (SMI of 60.7 cm^2^/m^2^), nor did he show myosteatosis (SMR of 46.1 HU).

**Figure S2.** Stratified PFS analysis based on the stage (A: stage III; B: stage IV) according to sarcopenia.

**Figure S3.** Stratified OS analysis based on the stage (A: stage III; B: stage IV) according to sarcopenia.

**Table S1.** Treatment and response characteristics for the whole population.
