# Supplementary figures and images for: Body composition derangements in lung cancer patients treated with first‐line pembrolizumab: A multicentre observational study
Source: J Cachexia Sarcopenia Muscle. 2024 Oct 22;15(6):2349–60. doi: 10.1002/jcsm.13568 (PMC11634481; doi:10.1002/jcsm.13568)

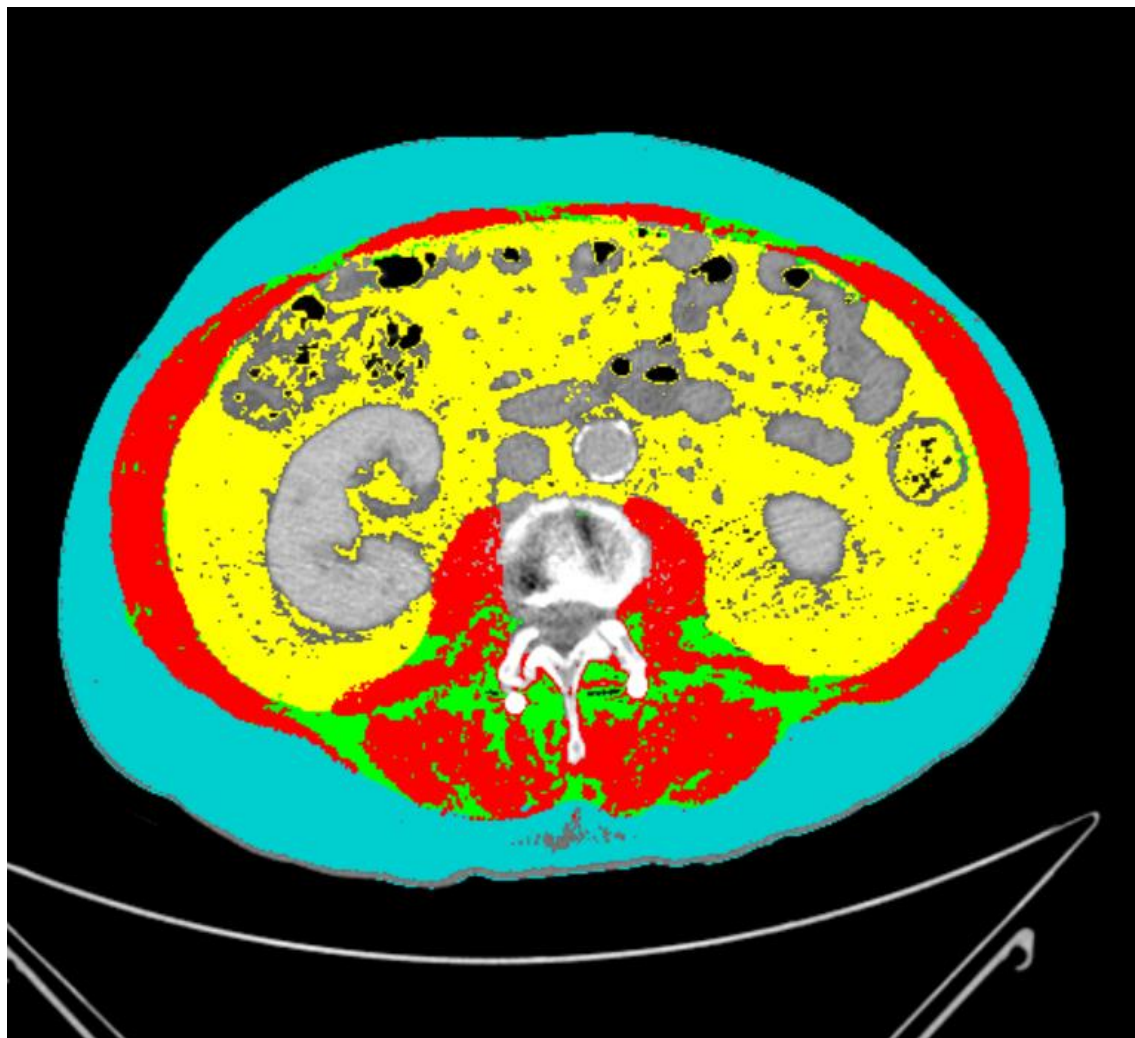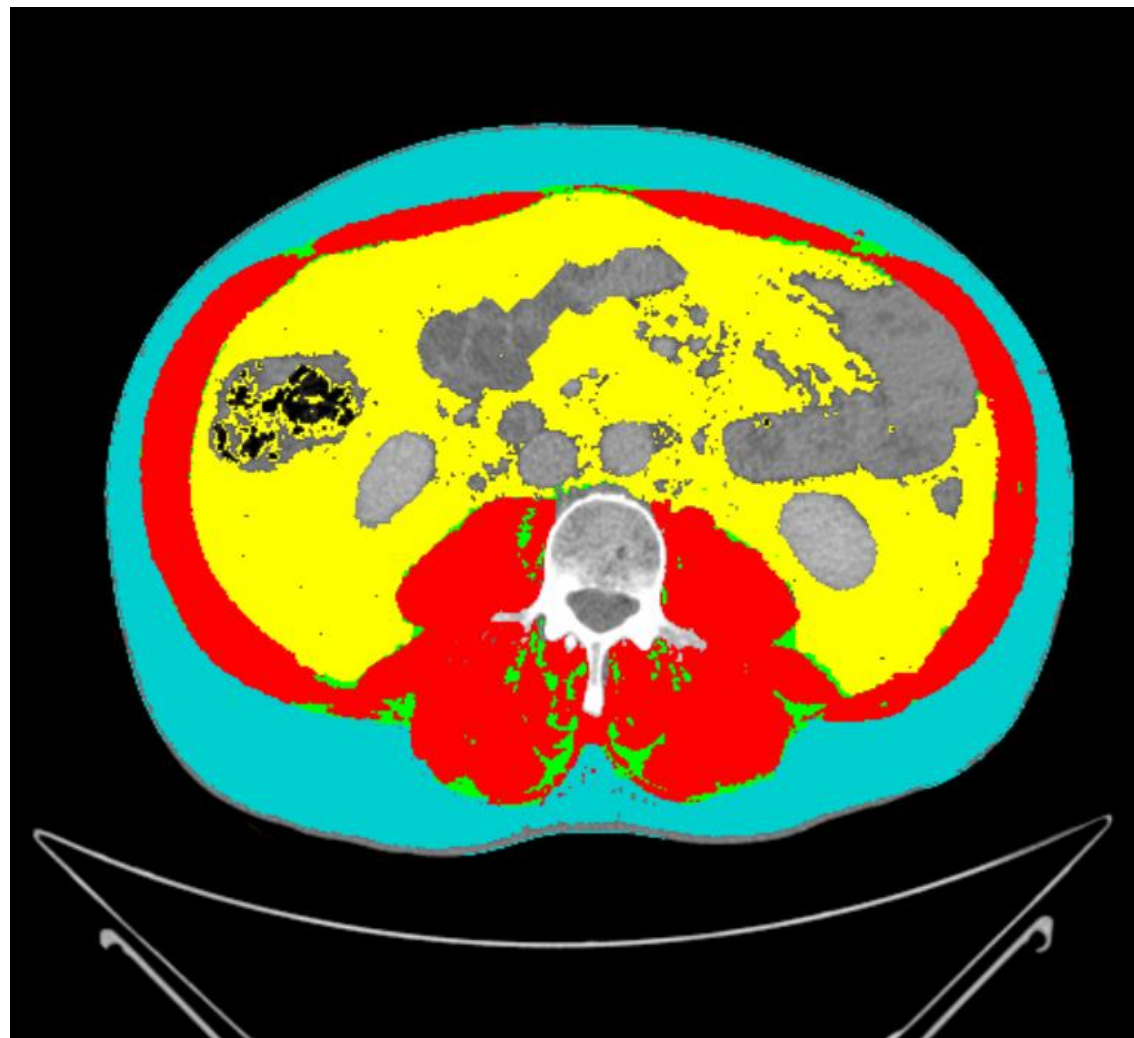

Supplement: Supplementary file 1 — Figure S1. Comparison of axial CT images between two male patients with the same BMI. Legend Supplementary Data 4: In red, lumbar skeletal muscle area (SMA) (cm2); in yellow, visceral adipose tissue area (VAT) (cm2); in teal: subcutaneous adipose tissue area (SAT) (cm2); in green, intermuscular adipose tissue area (IMAT) (cm2). Both patients had a BMI of 26.2 kg/m2. According to the established skeletal muscle index (SMI) cut‐off values, the first patient (left panel) reported sarcopenia, with a SMI value of 48 (<53) cm2/m2, and myosteatosis with a skeletal muscle radiodensity (SMR) of 32.1 (<33) HU. The second patient (right panel) did not show sarcopenia (SMI of 60.7 cm2/m2), nor did he show myosteatosis (SMR of 46.1 HU). [file JCSM-15-2349-s004.pdf]

## Slide 1
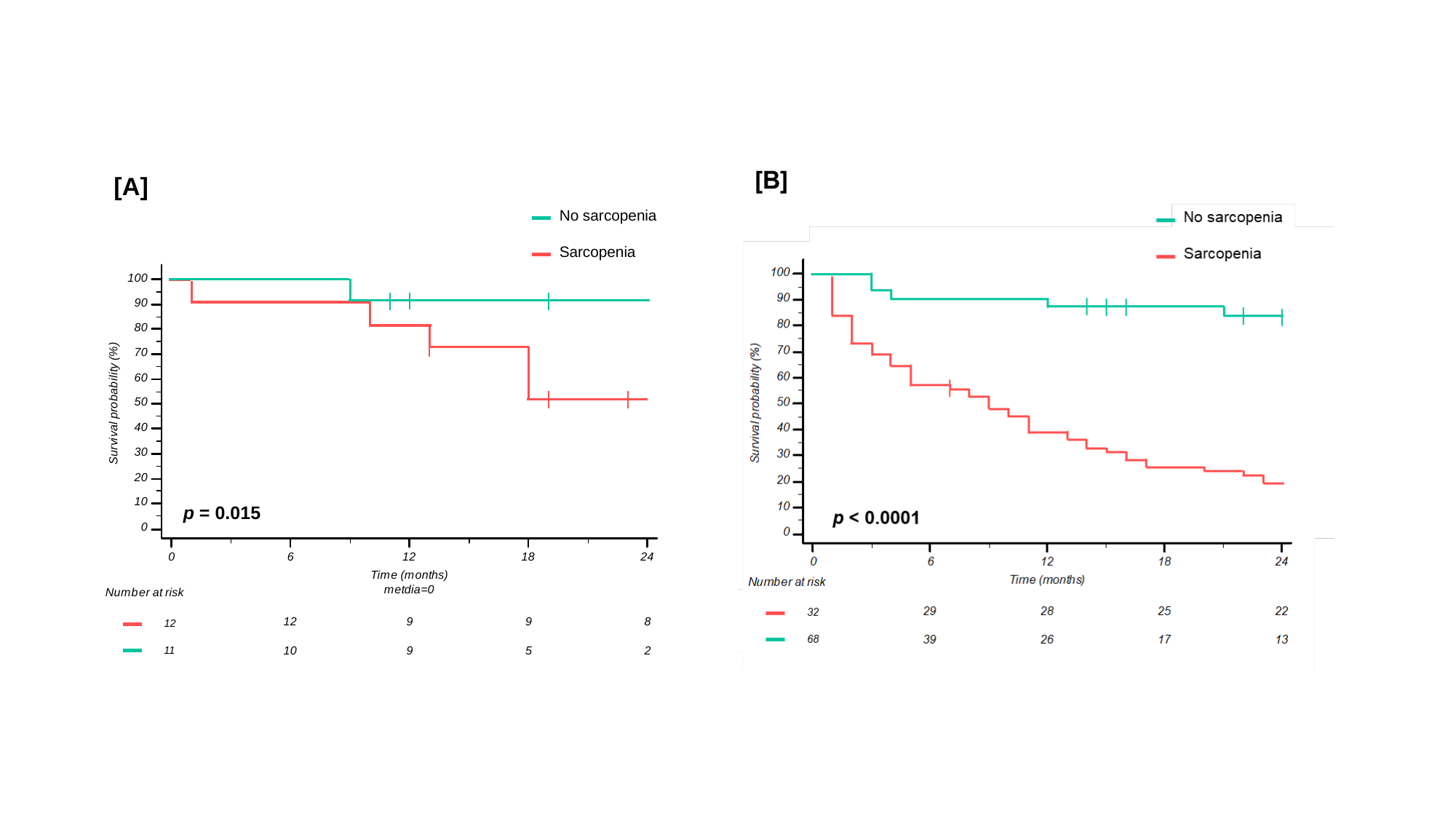

[A]
No sarcopenia
Sarcopenia
p = 0.015
12
11

Supplement: Supplementary file 3 — Figure S3. Stratified OS analysis based on the stage (A: stage III; B: stage IV) according to sarcopenia. [file JCSM-15-2349-s003.pptx]
