# Supplementary material for: Body composition derangements in lung cancer patients treated with first‐line pembrolizumab: A multicentre observational study
Source: J Cachexia Sarcopenia Muscle. 2024 Oct 22;15(6):2349–60. doi: 10.1002/jcsm.13568 (PMC11634481; doi:10.1002/jcsm.13568)
Supplement: Supplementary file 2 — Figure S2. Stratified PFS analysis based on the stage (A: stage III; B: stage IV) according to sarcopenia. [file JCSM-15-2349-s001.pptx]

## Slide 1
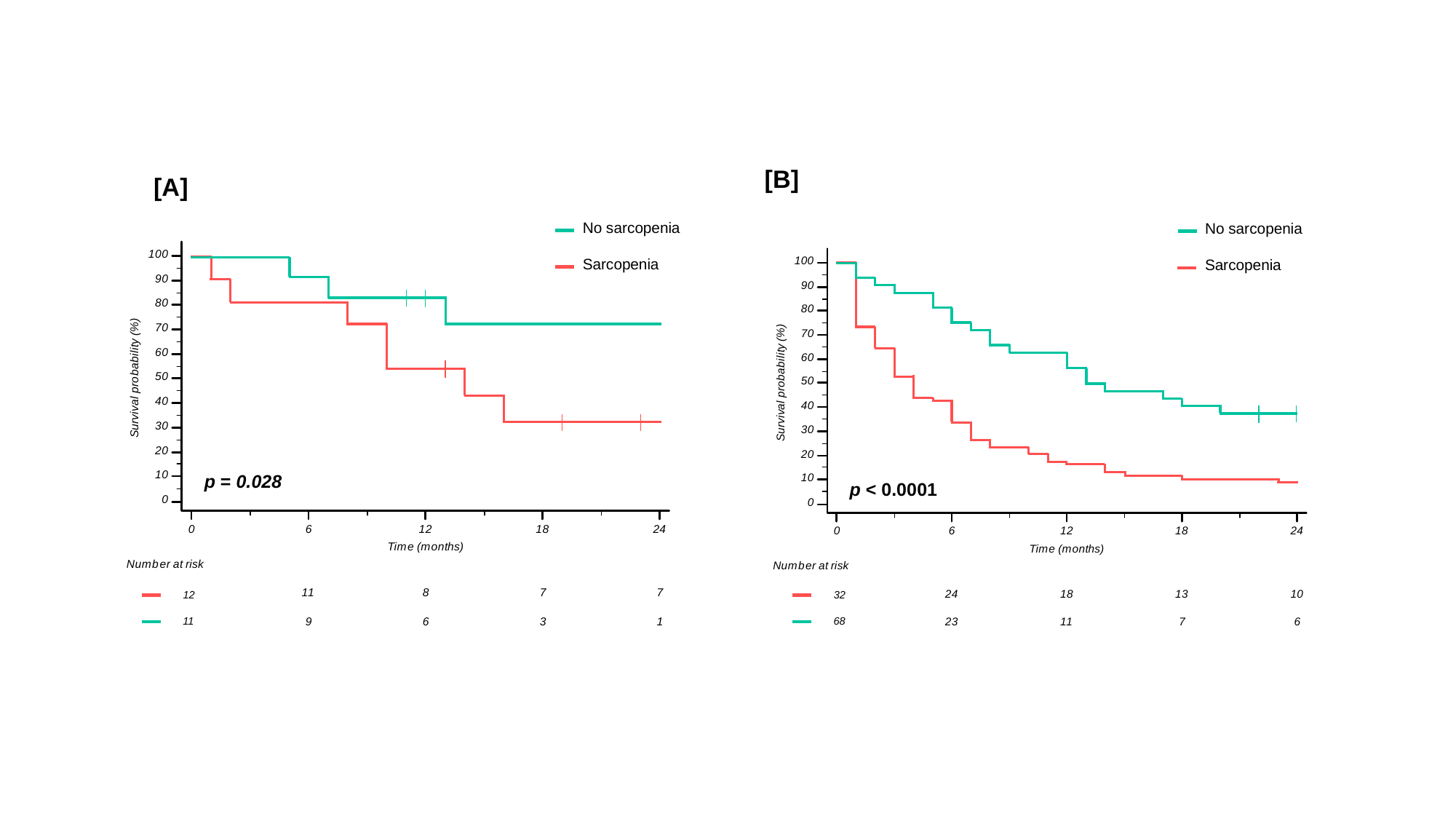

[B]
[A]
IV stadio
No sarcopenia
Sarcopenia
No sarcopenia
Sarcopenia
Other
P value=0,028
HR: 2,802 (CI95% 1,708-4,595)
P value=0,028
HR: 6,067 (CI95% 3,184-11,562)
p = 0.028
p < 0.0001
12
32
11
68
