# Supplementary material for: Body composition derangements in lung cancer patients treated with first‐line pembrolizumab: A multicentre observational study
Source: J Cachexia Sarcopenia Muscle. 2024 Oct 22;15(6):2349–60. doi: 10.1002/jcsm.13568 (PMC11634481; doi:10.1002/jcsm.13568)
Supplement: Supplementary file 4 — Table S1. Treatment and response characteristics for the whole population. [file JCSM-15-2349-s002.docx]

**Supplementary Table 1.** Treatment and response characteristics for the whole population.

| **Variables** | **Overall patients** | |
| --- | --- | --- |
|  | **N** | **%** |
| **Best response to immunotherapy** |  |  |
| CR | 6 | 4.5 |
| PR | 57 | 42.5 |
| SD | 23 | 17.2 |
| PD | 48 | 35.8 |
| **Progression-free survival (months)** | | |
| Median (95% CI) | 7.4 months (95%CI 5.6–9.2) | |
| **Overall survival (months)** | | |
| Median (95% CI) | 19.3 months (95%CI 12.7–26) | |

**Legend – Supplementary Table 1**: CR, complete response; PR, partial response; SD, stable disease; PD, progressive disease.
